# Association of Symptoms with Eating Habits and Food Preferences in Chronic Gastritis Patients: A Cross-Sectional Study

**DOI:** 10.1155/2020/5197201

**Published:** 2020-07-09

**Authors:** Yuan Li, Zeqi Su, Ping Li, Yicong Li, Nadia Johnson, Qi Zhang, Shihao Du, Huali Zhao, Kexin Li, Chi Zhang, Xia Ding

**Affiliations:** ^1^Dongzhimen Hospital, Beijing University of Chinese Medicine, Beijing 100029, China; ^2^Beijing Research Institute of Chinese Medicine, Beijing 100029, China; ^3^International School, Beijing University of Chinese Medicine, Beijing 100029, China; ^4^School of Traditional Chinese Medicine, Beijing University of Chinese Medicine, Beijing 100029, China; ^5^Institute of Basic Research in Clinical Medicine, China Academy of Chinese Medical Sciences, Beijing 100700, China

## Abstract

**Purpose:**

There is a lack of research on the relationship between symptoms and dietary factors of chronic gastritis (CG) patients, and the contribution of dietary management in relieving symptoms of CG patients has not attracted enough attention. This study aimed to identify the associations between different symptoms and dietary factors. *Patients and Methods*. All CG patients in this cross-sectional study were recruited from 3 hospitals in Beijing, China, from October 2015 to January 2016. Association Rule Mining analysis was performed to identify the correlations between gastrointestinal symptoms and dietary factors (including eating habits and food preferences), and subgroup analysis focused on gender differences.

**Results:**

The majority of patients (58.17%) reported that their symptoms were related to dietary factors. About 53% reported that they had the habit of “eating too fast,” followed by “irregular mealtimes” (29.66%) and “eating leftover food” (28.14%). Sweets (27.57%), spicy foods (25.10%), and meat (24.33%) were the most popular among all participants. Stomachache and gastric distention were the most common symptoms and were both associated with irregular mealtimes, irregular meal sizes, eating out in restaurants, meats, barbecue, fried foods, sour foods, sweets, snacks, and salty foods (support >0.05 and lift >1.0). Their most strongly associated factors were irregular meal sizes, barbecues, and snacks (lift >1.2). In addition, irregular mealtimes, salty foods, and sweet foods may be important diet factors influencing the symptoms in CG patients (support >0.05 and lift >1.0), as they were associated with almost all dyspeptic symptoms in the whole group and subgroup analyses. Furthermore, alcohol, barbecue, and spicy foods were associated with almost all symptoms for males (support >0.05 and lift >1.0), but sweets were the only dietary factor associated with all symptoms for females (support >0.05 and lift >1.0).

**Conclusion:**

This study has provided new data for the association of symptoms with eating habits and food preferences in CG patients. The role of individual daily management schemes, such as dietary or lifestyle programs, needs more attention.

## 1. Introduction

Chronic gastritis (CG) is one of the most common and insidious diseases in human beings; hundreds of millions of people worldwide suffer from this inflammatory condition to varying degrees and extent [1]. In China, the prevalence of CG based on endoscopic diagnosis is close to 90% [2]. Historical studies failed to demonstrate a significant association between CG and gastrointestinal symptoms, as CG does not always cause signs and symptoms. However, many CG patients in clinical practice mainly complain of gastrointestinal discomforts, such as stomachache, bloating, nausea, vomiting, and loss of appetite. In fact, in a national multicenter survey in China involving 8892 CG patients, 86.8% of patients presented with gastrointestinal symptoms, and about 40% of CG patients complained of more than one such symptom [3].

In recent years, the relationship between diet and disease has received more and more attention and has been increasingly exposed. In the cascading progression from CG to cancerization, the initial stage is associated with excessive salt intake and *Helicobacter pylori* (*H. pylori*) [4, 5], while the intake of sodium sulfite and sodium nitrate/sodium nitrite is the main risk factor during the intermediate stage, and the final stage has been found to be associated excessive salt intake and N-nitroso compound produced by nitrates in processed foods [6, 7]. In addition, studies reported that the risk of gastric cancer increased with high consumption of processed meat [8], barbecues [9], dry fish [10], and cooking oil [11]. The influence of vegetables and fruits against gastric carcinogenesis might be explained by the rich content of nutrients such as ascorbic acid, carotenoids, and *β*-carotene [12]. Despite reports that dietary supplementation with antioxidant micronutrients may interfere with the precancerous process, it is not currently endorsed as a therapy to decrease the risk of progression of gastric precancerous lesions due to an insufficient level of research evidence [13, 14].

Most studies highlight the dietary factors of patients already suffering from gastric cancer [15, 16], and few studies focus on their precursors. Moreover, dietary factors as a serious influence are largely underrated in symptomatic CG patients, and correlation analysis between different symptoms and dietary factors is insufficient. Although there is no clear evidence showing a causal relationship between certain dietary intake and the occurrence of CG symptoms, and there is no large clinical study on the efficacy of the dietary intervention, changes in dietary habits and lifestyle adjustment are part of the treatment of CG. The 2017 Chinese consensus on chronic gastritis suggested that individual adjustments in diet and lifestyle may be reasonable. Dietary and lifestyle changes, such as avoiding excessive coffee and alcohol consumption and long-term heavy smoking, are also frequently mentioned in clinical practice [17, 18].

Therefore, in this study, we focused on CG patients with gastrointestinal symptoms and aimed to identify the associations between different symptoms and dietary factors (including eating habits and food preferences). It may contribute to a more diverse approach to alleviating the symptoms of patients with CG, such as establishing a healthier diet pattern rather than relying solely on drugs, thereby improving the patients' quality of life.

## 2. Material and Methods

### 2.1. Participants and Recruitment

In this cross-sectional study, all participants were recruited from 3 hospitals (Dongzhimen Hospital Affiliated to Beijing University of Chinese Medicine, Dongfang Hospital Affiliated to Beijing University of Chinese Medicine, and Wangjing Hospital of China Academy of Chinese Medical Sciences) in Beijing, China from October 2015 to January 2016. Participants were selected based on the following inclusion criteria: (1) adults aged 18 years or above; (2) histological evaluation being performed according to the Updated Sydney System [19], which was graded according to the intensity of mononuclear inflammatory cellular infiltrates within the lamina propria: absent inflammation (Grade 0), mild inflammation (Grade 1), moderate inflammation (Grade 2), and severe inflammation (Grade 3); and (3) all consecutive patients with gastrointestinal symptoms for a week or longer. Exclusion criteria included (1) subjects who reported having other digestive system diseases such as peptic ulcer, pancreatitis, hepatitis, and cirrhosis; (2) serious heart, brain, liver, kidney, or hematopoietic system dysfunction or other serious diseases; (3) severe mental illness (such as schizophrenia, depression, and anxiety); (4) food or drug allergies, pregnancy, or breastfeeding; and (5) symptoms that occurred after taking prescription or over-the-counter drugs, especially aspirin or other pain relievers.

### 2.2. Ethics and Consent

This study protocol was reviewed and approved by the Medical Ethical Committee of Dongzhimen Hospital Affiliated to Beijing University of Chinese Medicine (No. ECPJ-BDY-2014-02) and performed in accordance with the principles of the Helsinki Declaration of 1975, as revised in 2008. After the survey had been fully explained, all participants provided written informed consent.

### 2.3. Data Collection

The CG patient self-report questionnaire's item pool referenced literature as previously reported [20–23], and the items finally included in the questionnaire were determined by multiple rounds of an expert consultation. The standardized questionnaire was composed of three sections: sociodemographic characteristics (including age, sex, educational level, height, and weight); current gastrointestinal symptoms (stomachache, gastric distention, hiccup, belching, acid reflux, heartburn, abdominal bloating, nausea, abdominal pain, borborygmus, vomiting, diarrhea, and early satiety, appetite, quantity of food, and symptom-related triggers); and dietary factors (including eating habits and food preferences). All subjects were asked to indicate how much they liked or disliked each food item by using the standard hedonic preference scale [24]. The scale, developed in 1957, is a fully anchored 9-point category scale. The scale ranges from 1 (dislike extremely) to 9 (like extremely), with a neutral point at 5 (neither like nor dislike). The interviewers judged the food preference of the patients in a unified manner. Scores less than or equal to 5 points were judged as “Yes,” and those greater than or equal to 6 points were judged as “No.” To reduce measurement bias, all interviewers were medical personnel trained to use standardized protocols to minimize interrater measurement bias. After obtaining consent, participants completed the paper-based survey individually or, if requested by participants, medical personnel administered the survey in an interview format by reading questions and documenting responses. Overall, each survey took approximately 15–30 min to complete. Confidentiality, authenticity and integrity of all the information gathered in the present study were highlighted and maintained at all levels of data management. Before data entry, double-checking of range and logic of every variable value was performed by primary researchers, and questionnaires with incomplete information were eliminated. Data were then double-entered into a dedicated database by data administrators to confirm that there was no input error.

### 2.4. Statistical Analysis

All analysis was run using *R* 3.5.0. We used the Association Rule Mining (ARM) analysis to identify the combinations of symptoms and dietary factors that cooccur in CG patients. We made use of the 4 common terms in ARM: (a) “association rule” is a combination of 2 items (e.g.,{dietary characteristic}⟶{symptom}) with an “antecedent” and a “consequent”; (b) “support” refers to how frequently the combinations appear in the data set; (c) “confidence” refers to the conditional probability of a symptom given that a participant has a certain dietary characteristic; (d) “lift” refers to the ratio of the confidence of the association rule to the expected confidence, assuming that the symptom was independent. The lift was considered the main measure of significance in this study. A value of “lift” greater than 1 indicates that the “association rule” appears more often together than expected, which could be interpreted as the antecedent factor ({dietary characteristic}) having a positive effect on the occurrence of the consequent ({symptom}). A value of “lift” less than 1 indicates that the “association rule” appears less often than expected, which means that the occurrence of the antecedent factor has a negative effect on the occurrence of the consequent. A lift value close to 1 indicates that the occurrence of the antecedent has little or no effect on the occurrence of the consequent conditions. Hence, the higher the lift, the higher the chance of cooccurrence of the consequent with the antecedent and the more significant the association. In our study, we eliminated all rules that have “lift” <1 (parameter setting: “support” >0.05, “lift” >1.0), and the results of ARM analyses are presented using summary tables and graphical visualizations showing the association rules of symptoms and dietary factors. In consideration of biological differences between males and females that could drive variations in dietary requirements, gender subgroup analysis was performed to compare the participation of males and that of females.

## 3. Results

### 3.1. Participant Characteristics

A total of 550 questionnaires were distributed, and 542 were returned. 526 forms were deemed eligible for this study after eliminating questionnaires with incomplete information for a final acceptance rate of 95.64%. Among the participants, 346 (65.8%) were female and 180 (34.2%) were male. The mean age of the sample was 49 years (SD ± 13.52) with approximately 40% of the participants between 19 and 44 years. The prevalence of *H. pylori* infection was found to be 47.15% in this sample, according to the result of the C-Urea Breath Test, biopsy urease testing, or histology within a month. Most patients (56.7%) had a normal body mass index (BMI), while the majority of the remainder had BMIs indicating that they were overweight (Table 1).

### 3.2. Self-Reported Symptoms and Precipitating Factors

**Figure 1:**
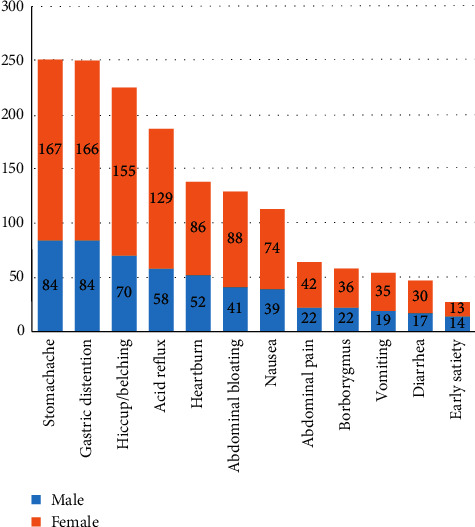
Symptoms ranking of the study population.

We found that the stomach was a common site of gastrointestinal symptoms in CG patients, reflected in Figure 1. Stomachache, gastric distension, and hiccup/belching were the three most common symptoms, accounting for 47.72%, 47.53%, and 42.78%, respectively. There was no statistical difference between each symptom stratified by gender (*P* > 0.05). From the perspective of contributing factors, the majority of patients (306, 58.17%) reported that their symptoms were related to dietary factors. The emotional state was also an important factor (197, 37.45%), especially for female participants. More than 40% of female patients believed that emotional factors affect their symptoms, 1.5 times higher compared with male patients (*P*=0.008). A small number of patients thought that environmental climate or fatigue was a critical factor.

### 3.3. Self-Reported Eating Habits and Food Preferences

The data on eating habits and food preferences are shown in Table 2. 278 (52.85%) of the subjects reported having the habit of “eating too fast,” and “irregular mealtimes” and “eating leftover food” were reported by 29.66% and 28.14%, respectively. Sweets (27.57%), spicy foods (25.10%), and meat (24.33%) were the most popular food preferences among all participants. When comparing genders, we found that men were more likely to eat leftover food and eat out in restaurants than women (*P* < 0.05) but had no significant differences in other eating habits. Compared with females, male participants preferred meat, barbecue, and alcohol (*P* < 0.05) while female participants preferred sour foods more than males (*P* < 0.05).

### 3.4. Association Rules of Symptoms and Dietary Factors

**Figure 2:**
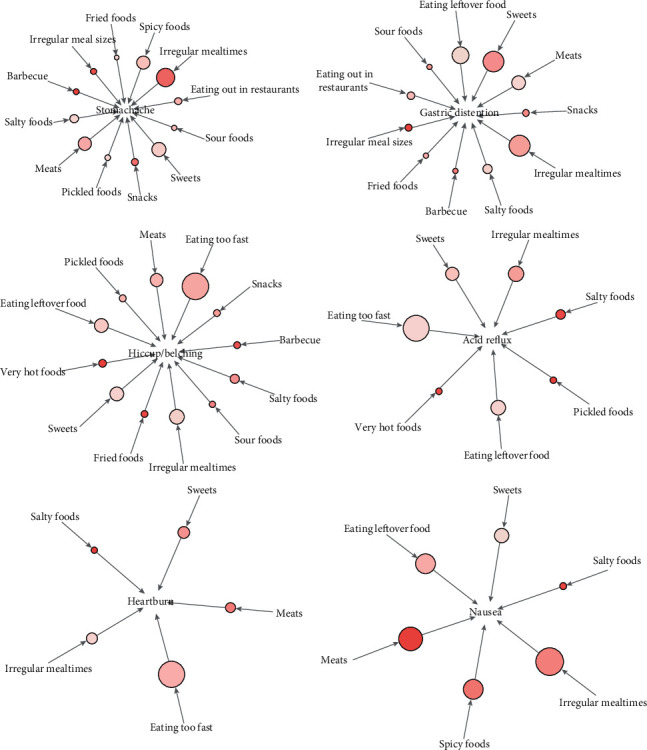
ARM analysis of symptoms and dietary factors. The arrows indicate the relationship between rules, and directions of the arrows indicate antecedent and consequent items. The size of the circle represents the level of “support” associated with the rule (the larger the circle, the larger the support value), and the color represents the level of “lift” with the rule (the darker the color, the larger the lift value).

We focused on the symptoms (frequency exceeds 100) that occur in the stomach and conducted ARM analysis on symptoms and dietary factors to explore their correlations and provide more illumination in clinical practice. Visualization of the association rules is shown in Figure 2. 12 dietary factors had effective association rules with a stomachache (support >0.05 and lift >1.0). Irregular mealtimes, sweets, spicy foods, and meats occurred more frequently in these association rules (support >0.1 and lift >1.0), indicating that more stomachache patients were accompanied by these dietary factors. The strongest associations were found between a stomachache and barbecue (lift = 1.305), irregular mealtimes (lift = 1.272), irregular meal sizes (lift = 1.236), and snacks (lift = 1.217), which can be interpreted as a higher likelihood of stomachache given the above dietary factors. The detailed results are shown in Table 3.

We also found that there were some similar dietary factors between different symptoms, such as stomachache and gastric distention, which were both related to irregular mealtimes, irregular meal sizes, eating out in restaurants, meats, barbecue, fried foods, sour foods, sweets, snacks, and salty foods (support >0.05 and lift >1.0). Irregular meal sizes, barbecues, and snacks were the most strongly associated factors (lift >1.2). Hiccup/belching and acid reflux were both related to eating too fast, irregular mealtimes, eating leftover food, very hot foods, sweets, pickled foods, and salty foods (support >0.05 and lift >1.0), with consumption of very hot foods as the strongest dietary characteristic (lift >1.2). Heartburn and nausea were both related to irregular mealtimes, meats, sweets, and salty foods (support >0.05 and lift >1.0), especially meats and salty foods (lift >1.2).

All six symptoms were noticeably associated with irregular mealtimes, sweets, and salty foods (support >0.05 and lift >1.0). In particular, among these effective association rules, the combination of “irregular mealtimes ⟶ nausea,” “sweets ⟶ gastric distension,” “sweets ⟶ stomachache,” and “salty foods ⟶ nausea” revealed stronger cooccurrence (lift >1.2).

### 3.5. Subgroup Analysis Based on Gender Differences

**Figure 3:**
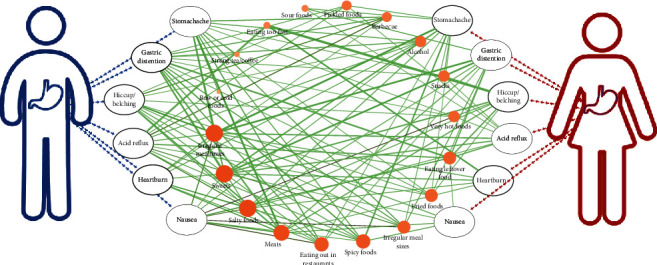
The relationship between symptoms with dietary factors stratified by gender. The size of the orange circle represents the number of related symptoms (the larger the circle, the larger the number). The size of the green line represents the level of “support” associated with the rule (the thicker the line, the larger the support value), and the color represents the level of “lift” with the rule (the darker the color, the larger the lift value).

We also found some very interesting phenomena when we discussed the relationship between symptoms and dietary factors in different gender groups (Figure 3, Table 4). Although in the aforementioned whole group analysis we found that these three dietary factors above (alcohol, strong tea/coffee, and raw/cold food) were not antecedent factors for any symptoms, effective association rules were found in the male participant group, especially alcohol, which was associated with all symptoms (support >0.05 and lift >0.1). Besides alcohol and eating out in restaurants, preference for barbecue and spicy foods was also associated with almost all symptoms (support >0.05 and lift >0.1) in male patients. However, these correlations were hardly established in female patients.

In general, the dietary factors that may affect the symptoms in female patients were more concentrated, while the “dietary factors ⟶ symptom” combination in men was more diversified. For example, stomachache, which has 10 effective association rules in female patients, is mostly associated with irregular mealtimes (lift = 1.266); however, 13 effective association rules were found in male patients and are mostly associated with multiple factors of raw or cold foods (lift = 1.607), snacks (lift = 1.378), and barbecue (lift = 1.349). Finally, irregular mealtimes and salty and sweet foods were associated with most symptoms in both men and women (support >0.05 and lift >0.1). Sweets specifically were the only dietary factors associated with all symptoms in females.

## 4. Discussion

In our study, approximately 58% of CG patients believe that their symptoms are affected by dietary factors, but it is difficult for them to point out the specific relationships and manage their diet. As for the remaining patients who believe that there is no association between diet and symptoms, is it possible that identifying the potential long-term effects of dietary habits is too difficult for patients on their own? This may, in fact, reflect the difficulty in assessing a potential relationship, rather than an actual lack of an association. Therefore, we hope to find some possible connections through this survey and provide a reference for clinical practice and patient self-management. The present study revealed that many gastrointestinal symptoms of CG patients correlate with unhealthy eating habits and food preferences, among which irregular mealtimes, salty foods, and sweets are the strongest risk factors. In addition, there was a difference between genders in the relationship between dietary factors and symptoms. Male patients likely need to limit the consumption of alcohol, barbecue, and spicy foods and reduce the frequency of eating out. Female patients especially need to pay attention to controlling the intake of sweets.

In this cross-sectional analysis of CG patients, irregular mealtimes and preference for sweet or salty foods were associated with all symptoms in the whole group analysis. Furthermore, in the gender subgroup analysis, these three factors were associated with most symptoms in both men and women, which again suggested that these three dietary factors may be the key to affecting symptoms in CG patients. Therefore, we further discuss the above three core factors in combination with modern medical research.

Eating at irregular mealtimes is a key factor especially associated with stomachache and nausea. Epidemiological analysis has demonstrated that circadian dysfunction leads to a significantly increased risk for gastrointestinal symptoms, especially abdominal pain [25, 26]. However, modern lifestyles frequently disrupt circadian rhythm, which may be an important factor in the current high incidence of gastrointestinal diseases. Sugar and salt are indispensable flavorings, but in recent years, a growing body of research has shown that excessive intake can increase the risk of multiple chronic diseases. The ingestion of sugar alcohols is associated with increased levels of colonic fermentation and a dose-dependent increase in gastrointestinal symptomatology, including colic, flatus, and borborygmi [27]. Many studies have demonstrated that high-intensity sweeteners can alter the gut microbiota, especially the gut glucose tolerance of the intestinal flora, and promote the development of chronic inflammation [28, 29]. Salt especially affects gastroesophageal reflux-related symptoms like nausea, heartburn, and acid reflux. The association between reflux and salt intake has been reported in previous studies; other frequently mentioned diet-related risk factors include, but are not limited to, alcohol, smoking, rapid food intake, irregular food intake, high-fat diets, spicy foods, acidic foods, coffee, and chocolate [30–34]. Although most of these factors were also found in our study, we believe that salt may be the more critical factor, especially from the perspective of patients with multiple symptoms. In addition, salt has long been known for its adverse health effects, and high levels of sodium chloride damage the walls of the stomach and augment the expression of proinflammatory enzymes and cytokines in gastric mucosa with *H. pylori* infection, cause cell death, and induce regenerative cell proliferation, resulting in inflammation and atrophy [35].

In addition, the differences between men and women concerning eating characteristics and their associations with symptoms are noteworthy. Previous studies on the effects of diet on health have been mostly reported from a disease perspective, and there are relatively few reports on the differences in diet habits between the different genders. In our study's gender subgroup analysis, there are some obvious differences in dietary factors and symptoms between males and females. In addition to the three core factors mentioned above, male patients also need to control alcohol intake, eating out in restaurants, and their preference for barbecue and spicy foods, but female patients may especially need to pay attention to controlling the intake of sweets. On the one hand, this does in fact reflect differences in eating habits between males and females. On the other hand, physiological differences between men and women may also result in differences in food sensitivity, digestion, and metabolism. Therefore, a stratified analysis of gender is necessary, as this particular problem needs to be considered and deserves the attention of future research.

Overall, the results of this study suggest a significant correlation between eating habits and symptoms, which is consistent with previous research evidence. As we know, CG is a multistep, progressive, and life-long inflammatory condition, and the symptoms are complex and varied. Considering that there is still a lack of effective medicines for different symptoms, more diverse care models should be explored, including patient self-management skills like daily diet rather than relying solely on drug therapy. CG can be painful, but there are some changes you can make that may markedly improve your symptoms and prevent the condition from getting worse. Eating healthy foods and avoiding those that can trigger inflammation, such as we mentioned above, can both lessen the pain and other symptoms associated with gastritis as well as prevent irritation of the stomach lining from getting worse. Many people who suffer from CG know exactly which food items exacerbate their symptoms, but many do not. You can create a food diary and write down everything you eat or drink and note the exact date/time you eat it for some weeks. Simultaneously, write down any time you experience gastritis symptoms. That may help you find a personalized and healthy diet that suits you.

Nevertheless, inevitably, there are flaws and deficiencies in this study. Firstly, the participants in this study were limited to the Beijing area, and the sample size was relatively small. Therefore, the conclusions may be generalized only to CG patients in Beijing, and the results still require a larger sample size and multicenter study in order to be further clarified and validated. Moreover, there is no comparison group within the selected sample, all results are merely generalization to part of the population that underwent endoscopy for a logical reason and has CG in their histology. We will exclude CG patients and compare the results with existing results, hoping to obtain more beneficial evidence. The third limitation is the lack of management of confounders. We focused primarily on dietary factors, and other potential influencing factors (such as *H. pylori*) are not included in the outcome analysis. Lastly, the cross-sectional study design and AMR analysis methods can only be used to identify combinations of dietary factors and symptoms and cannot establish any causal links, which warrant further investigation. Hence, a longitudinal study with a focus on trajectories of the development of these conditions may shed more light on causality. Despite these limitations, the major strength of this report lies in practical value for the management of CG patients and provides some references for future research. We are continuing to collect data, and based on this research, we have optimized our questionnaires and program coverage to provide better evidence.

## 5. Conclusion

Collectively, the current study provided evidences that eating habits and food preferences were indeed associated with gastrointestinal symptoms. It is beneficial and necessary to adjust the diet habits of CG patients, especially to control the intake of sweets, salty foods, meats, spicy foods, and fried foods. In addition, the formation of regular eating habits is a very important factor. Moreover, focusing on different gender groups of CG patients, some specific dietary factors should be paid more attention, which would be of great significance in the control of CG disease. However, because the present research was based on cross-sectional data, further research should focus on longitudinal data to assess the trajectories of the dynamic transition between diet and gastrointestinal symptoms.

## Figures and Tables

**Table 1:** Descriptive characteristics of the study population (*N* = 526).

Characteristics	Participants (*N* (%))
*Gender*	
Male	180 (34.2%)
Female	346 (65.8%)

*Age, y*	
19∼44	202 (38.4%)
45∼59	186 (35.4%)
60∼74	126 (24.0%)
75∼89	12 (2.2%)

*Education*	
Primary (≤9 y)	168 (32%)
Secondary (9–12 y)	154 (29%)
Some college (13–17 y)	191 (36%)
College graduate or higher (≥18 y)	13 (3%)

*Helicobacter pylori*	
(+)	248 (47.15%)
(−)	278 (52.85%)

*BMI, kg/m* ^2^	
Underweight (<18.5)	39 (7.4%)
Normal weight (18.5∼23.9)	298 (56.7%)
Overweight (24∼28)	149 (28.3%)
Obesity (>28.0)	40 (7.6%)

**Table 2:** CG patients' self-reported dietary factors.

Dietary factors	*N* (*n* = 526)	Male (*n* = 180)	Female (*n* = 346)	Comparison between genders	
				Χ^*2*^	*P*
Eating habits					
Eating too fast	278 (52.85%)	103 (57.22%)	175 (50.58%)	2.098	0.175
Irregular mealtimes	156 (29.66%)	48 (26.67%)	108 (31.21%)	1.174	0.326
Eating leftover food	148 (28.14%)	61 (33.89%)	87 (25.14%)	4.478	0.044
Eating out in restaurants	74 (14.07%)	34 (18.89%)	40 (11.56%)	5.260	0.031
Irregular meal sizes	56 (10.65%)	24 (13.33%)	32 (9.25%)	2.077	0.196

Food preferences					
Sweets	145 (27.57%)	47 (26.11%)	98 (28.32%)	0.290	0.663
Spicy foods	132 (25.10%)	36 (20.00%)	96 (27.75%)	3.779	0.066
Meats	128 (24.33%)	58 (32.22%)	70 (20.23%)	9.246	0.003
Salty foods	88 (16.73%)	30 (16.67%)	58 (16.76%)	0.001	1.000
Very hot foods	65 (12.36%)	21 (11.67%)	44 (12.72%)	0.121	0.836
Pickled foods	64 (12.17%)	19 (10.56%)	45 (13.01%)	0.665	0.500
Snacks	62 (11.79%)	14 (7.78%)	48 (13.87%)	4.230	0.056
Sour foods	56 (10.65%)	11 (6.11%)	45 (13.01%)	5.917	0.022
Fried foods	56 (10.65%)	20 (11.11%)	36 (10.40%)	0.062	0.920
Barbecue	53 (10.08%)	27 (15.00%)	26 (7.51%)	7.322	0.011
Strong tea/coffee	43 (8.17%)	18 (10.00%)	25 (7.23%)	1.214	0.350
Raw or cold foods	39 (7.41%)	12 (6.67%)	27 (7.80%)	0.223	0.767
Alcohol	36 (6.84%)	28 (15.56%)	8 (2.31%)	32.571	<0.001
Carbonated beverages	12 (2.28%)	5 (2.78%)	7 (2.02%)	0.302	0.555

**Table 3:** ARM results of all symptoms with dietary factors.

		Eating too fast	Irregular mealtimes	Irregular meal sizes	Eating leftover food	Eating out in restaurants	Meats	Barbecue	Fried foods	Very hot foods	Alcohol	Spicy foods	Sour foods	Sweets	Snacks	Pickled foods	Salty foods	Strong tea/coffee	Raw or cold foods
Stomachache	Support	—	**0.175**	**0.065**	—	**0.074**	**0.129**	**0.063**	**0.053**	—	—	**0.129**	**0.055**	**0.139**	**0.068**	**0.061**	**0.084**	—	—
	Lift	—	**1.236**	**1.272**	—	**1.104**	**1.113**	**1.305**	**1.04**	—	—	**1.080**	**1.085**	**1.055**	**1.217**	**1.048**	**1.048**	—	—
Gastric distention	Support	—	**0.162**	**0.070**	**0.137**	**0.072**	**0.118**	**0.059**	**0.057**	—	—	—	**0.059**	**0.158**	**0.068**	—	**0.082**	—	—
	Lift	—	**1.146**	**1.390**	**1.024**	**1.080**	**1.019**	**1.231**	**1.127**	—	—	—	**1.165**	**1.204**	**1.221**	—	**1.028**	—	—
Hiccup/Belching	Support	**0.243**	**0.129**	—	**0.124**	—	**0.114**	**0.051**	**0.055**	**0.065**	—	—	**0.051**	**0.120**	**0.055**	**0.055**	**0.080**	—	—
	Lift	**1.076**	**1.019**	—	**1.027**	—	**1.096**	**1.191**	**1.211**	**1.223**	—	—	**1.127**	**1.016**	**1.093**	**1.059**	**1.116**	—	—
Acid reflux	Support	**0.188**	**0.114**	—	**0.108**	—	—	—	—	**0.053**	—	—	—	**0.101**	—	**0.053**	**0.072**	—	—
	Lift	**1.001**	**1.082**	—	**1.083**	—	—	—	—	**1.212**	—	—	—	**1.028**	—	**1.231**	**1.215**	—	—
Heartburn	Support	**0.156**	**0.082**	—	—	—	**0.078**	—	—	—	—	—	—	**0.086**	—	—	**0.059**	—	—
	Lift	**1.124**	**1.051**	—	—	—	**1.221**	—	—	—	—	—	—	**1.183**	—	—	**1.342**	—	—
Nausea	Support	—	**0.082**	—	**0.070**	—	**0.076**	—	—	—	—	**0.070**	—	**0.062**	—	—	**0.051**	—	—
	Lift	—	**1.283**	—	**1.164**	—	**1.455**	—	—	—	—	**1.305**	—	**1.060**	—	—	**1.428**	—	—

**Table 4:** ARM results of all symptoms with dietary factors stratified by gender.

		Eating too fast	Irregular mealtimes	Irregular meal sizes	Eating leftover food	Eating out in restaurants	Meats	Barbecue	Fried foods	Very hot foods	Alcohol	Spicy foods	Sour foods	Sweets	Snacks	Pickled foods	Salty foods	Strong tea/coffee	Raw or cold foods
*Female Patients*																			
Stomachache	Support	—	0.191	0.055	—	0.058	—	—	0.052	—	—	0.147	0.072	0.142	0.078	0.069	0.087	—	—
	Lift	—	1.266	1.230	—	1.036	—	—	1.036	—	—	1.101	1.151	1.036	1.165	1.105	1.072	—	—
Gastric distention	Support	—	0.182	0.064	0.121	0.058	0.098	—	0.055	—	—	—	0.078	0.168	0.078	—	0.081	—	—
	Lift	—	1.216	1.433	1.006	1.042	1.012	—	1.100	—	—	—	1.251	1.234	1.172	—	1.006	—	—
Hiccup/Belching	Support	0.263	—	—	0.121	—	0.101	—	0.055	0.069	—	—	—	0.133	0.067	—	0.081	—	—
	Lift	1.161	—	—	1.078	—	1.116	—	1.178	1.218	—	—	—	1.048	1.070	—	1.078	—	—
Acid reflux	Support	—	0.127	—	0.104	—	—	—	—	0.055	—	—	0.052	0.113	0.055	0.055	0.067	—	—
	Lift	—	1.093	—	1.110	—	—	—	—	1.158	—	—	1.073	1.067	1.062	1.133	1.064	—	—
Heartburn	Support	0.153	0.081	—	—	—	0.061	—	—	—	—	—	—	0.090	—	—	0.061	—	—
	Lift	1.219	1.043	—	—	—	1.207	—	—	—	—	—	—	1.273	—	—	1.457	—	—
Nausea	Support	—	0.078	—	0.055	—	0.064	—	—	—	—	0.075	—	0.064	—	—	—	—	—
	Lift	—	1.169	—	1.021	—	1.470	—	—	—	—	1.266	—	1.050	—	—	—	—	—

*Male Patients*																			
Stomachache	Support	—	0.144	0.083	—	0.106	0.194	0.094	0.056	0.056	0.083	0.094	—	0.133	0.050	—	—	0.056	0.050
	Lift	—	1.161	1.340	—	1.198	1.293	1.349	1.071	1.020	1.148	1.012	—	1.094	1.378	—	—	1.191	1.607
Gastric distention	Support	—	—	0.083	0.167	0.100	0.156	0.089	0.061	0.056	0.089	0.117	—	0.139	0.050	0.061	0.083	0.050	—
	Lift	—	—	1.339	1.054	1.135	1.035	1.27	1.179	1.020	1.225	1.25	—	1.140	1.378	1.241	1.071	1.071	—
Hiccup/Belching	Support	—	0.144	0.061	—	0.078	0.139	0.089	0.056	0.056	0.061	0.083	—	—	—	0.050	0.078	—	—
	Lift	—	1.393	1.179	—	1.059	1.108	1.524	1.289	1.225	1.010	1.071	—	—	—	1.218	1.200	—	—
Acid reflux	Support	0.189	0.089	0.067	0.117	0.061	—	0.083	0.050	—	0.067	0.072	—	—	—	0.050	0.083	—	—
	Lift	1.024	1.035	1.552	1.068	1.004	—	1.724	1.397	—	1.33	1.121	—	—	—	1.470	1.552	—	—
Heartburn	Support	—	0.083	—	—	0.067	0.111	0.050	—	—	0.061	0.078	—	0.078	—	—	0.056	—	—
	Lift	—	1.082	—	—	1.222	1.194	1.154	—	—	1.360	1.346	—	1.031	—	—	1.154	—	—
Nausea	Support	—	0.089	0.061	0.100	0.078	0.100	—	—	0.056	0.050	0.061	—	0.061	—	—	0.061	—	—
	Lift	—	1.539	2.115	1.362	1.901	1.432	—	—	2.198	1.484	1.080	—	1.080	—	—	1.692	—	—

## Data Availability

The data used to support the findings of this study are included within the article.
